# Cost-Effectiveness of Early vs Delayed Belimumab Treatment for Systemic Lupus Erythematosus

**DOI:** 10.1001/jamanetworkopen.2025.60167

**Published:** 2026-02-19

**Authors:** Sabrina Hundal, Julian Cappelli, Christopher Sjöwall, Mohamed Osman, Zahi Touma, Ioannis Parodis, Stephanie R. Goldberg, Elena Netchiporouk

**Affiliations:** 1 Department of Medicine, University of Calgary, Calgary, Alberta, Canada; 2 Division of Experimental Medicine, McGill University Health Centre, Montreal, Quebec, Canada; 3 Department of Health Research Methods, McMaster University, Hamilton, Ontario, Canada; 4 Department of Biomedical and Clinical Sciences, Division of Inflammation and Infection, Linköping University, Linköping, Sweden; 5 Department of Rheumatology, University of Alberta, Edmonton, Alberta, Canada; 6 Schroeder Arthritis Institute, Krembil Research Institute, University Health Network, Toronto, Ontario, Canada; 7 Centre for Prognosis Studies in Rheumatic Diseases, University of Toronto Lupus Clinic, Toronto Western Hospital, Toronto, Ontario, Canada; 8 Division of Rheumatology, Department of Medicine Solna, Karolinska Institutet, Karolinska University Hospital, and Center for Molecular Medicine, Stockholm, Sweden; 9 Department of Rheumatology, Faculty of Medicine and Health, Örebro University, Örebro, Sweden.; 10 Department of Surgery, Mary Washington Healthcare, Fredericksburg, Virginia; 11 Division of Dermatology, McGill University Health Centre, Montreal, Quebec, Canada

## Abstract

**Importance:**

Belimumab is a biologic therapy for active, autoantibody-positive systemic lupus erythematosus (SLE) that has been shown to reduce disease activity, flare frequency, and glucocorticoid use, thereby preventing organ damage. Belimumab is typically reserved for refractory disease, but emerging evidence suggests earlier initiation may lead to higher response rates, greater achievement of remission or low disease activity, and further reduction in glucocorticoid use.

**Objective:**

To evaluate the economic and health-related outcomes of early vs delayed belimumab initiation for biologic-naive patients with clinically active SLE, comparing medical costs and quality-adjusted life-years (QALYs).

**Design, Setting, and Participants:**

This economic evaluation using cost-utility analysis from a US payer perspective was conducted with a Markov model with monthly cycles over a 15-year horizon and was informed by studies published from 2013 to 2025, identified through a targeted literature review. Biologic-naive adult SLE patients with active disease (SLE Disease Activity Index 2000 score >0) were included. Patients began in a pretreatment state and transitioned monthly between 5 health states: complete response (per SLE Responder Index-4 [SRI-4]), partial response, nonresponse (failure to meet SRI-4 accompanied by flare or treatment-emergent adverse event), no treatment (standard of care), and death.

**Exposure:**

Early (≤2 years of disease duration) or delayed (after failure of standard therapy) initiation of intravenous belimumab.

**Main Outcomes and Measures:**

Primary outcomes included total direct medical costs, QALYs, incremental cost-effectiveness ratio (ICER), and incremental net monetary benefit (INMB). The 95% uncertainty intervals (UIs) for incremental costs, QALYs, and INMB were calculated from the 2.5 and 97.5 percentiles of the probabilistic sensitivity analyses.

**Results:**

The modeled cohort included 1000 adults (912 female [91.2%]) with a mean (SD) age of 41 (11) years at belimumab initiation. Early initiation provided an additional 0.30 (95% UI, −0.42 to 1.39) QALYs at an incremental cost of –$126 337.12 (95% UI, –$910 010.39 to $168 383.94) per patient relative to delayed initiation, yielding a favorable ICER of –$421 123.73 per QALY. At a $50 000 per QALY threshold, the mean INMB was $141 337.12 (95% UI, –$157 997.53 to $925 019.51), with early initiation preferred in 81.3% of simulations (8125 of 10 000 simulations). Deterministic sensitivity analyses identified time horizon (INMB range, $6351.07 for a 1-year horizon to $156 497.12 for a lifetime horizon) and SRI-4 response odds ratio (INMB range, $69 741.68 for an odds ratio of 1.08 to $209 591.09 for an odds ratio of 3.47) as the most influential parameters.

**Conclusions and Relevance:**

In this economic evaluation of early vs delayed belimumab in biologic-naive patients with active SLE, early initiation was associated with improved health outcomes and reduced costs. These findings support earlier clinical adoption and reconsideration of reimbursement criteria to reflect long-term value.

## Key Points

**Question:**

Is early belimumab initiation associated with higher cost-effectiveness than delayed initiation for biologic-naive adult patients with clinically active systemic lupus erythematosus?

**Findings:**

This economic evaluation using cost-utility analysis and integrated existing evidence on early and delayed belimumab initiation found that early initiation was more cost-effective. Using a Markov model over a 15-year horizon, early belimumab was associated with a gain of 0.30 quality-adjusted life-years and a cost savings of $126 337.12 per patient relative to delayed initiation, making it the dominant choice.

**Meaning:**

These findings suggest that early belimumab may offer greater value by improving health outcomes while reducing medical costs, supporting reconsideration of reimbursement criteria.

## Teaser

This economic evaluation compares economic and health-related outcomes of early vs delayed belimumab initiation for patients with clinically active systemic lupus erythematosus.

## Introduction

Systemic lupus erythematosus (SLE) is a chronic autoimmune disease affecting more than 3.4 million individuals worldwide.^1^ It is marked by relapsing-remitting inflammation, multiorgan involvement, and significant morbidity.^1^ Standard SLE therapy includes glucocorticoids, antimalarials, and immunosuppressants.^2^ While antimalarials are well tolerated, they are not universally effective.^2^ Prolonged glucocorticoid and immunosuppressant use is associated with cumulative toxic effects, underscoring the need for safer, more effective options.^3^ Although these conventional therapies carry relatively low drug acquisition costs, up to one-third of patients fail to achieve complete disease control, leaving them vulnerable to flares and damage accrual.^4^

Belimumab, a B-lymphocyte stimulator inhibitor, is the first biologic approved for SLE and has demonstrated efficacy in reducing disease activity, flare rates, and glucocorticoid exposure.^5,6^ While initially reserved for use as a late line of therapy due to its acquisition cost, evolving guidelines—including the 2023 European Alliance of Associations for Rheumatology update—now recognize its role earlier in the treatment course, particularly when glucocorticoid tapering is unsuccessful, or organ damage risk is high.^2^ Data increasingly suggest that early belimumab initiation (within 1-2 years of disease duration) leads to faster and more durable clinical responses, greater achievement of remission or low disease activity, and reduction in long-term damage.^7,8,9^

These findings highlight the health economic implications of early belimumab use amid rising biologic drug costs. Despite increasing evidence supporting earlier biologic therapy, current reimbursement frameworks continue to favor delayed initiation after failure of standard immunosuppressants. In the absence of published cost-utility analyses comparing early vs delayed belimumab initiation, we evaluated its cost-effectiveness from the US payer perspective.

## Methods

### Study Design and Ethics

This economic evaluation used cost-utility analysis to compare early vs delayed intravenous belimumab in biologic-naive adults with clinically active SLE. The analysis was performed in 2025 from the US health care system perspective and followed the Consolidated Health Economic Evaluation Reporting Standards (CHEERS) reporting guideline.^10^ A glossary of commonly used health economic terms can be found in the Table. This study exclusively used previously published, deidentified aggregate data and did not involve human participants. In accordance with federal regulations and institutional policy, the study was exempt from institutional review board oversight.

**Table.** Glossary of Health Economic Terms

Term	Definition
CUA	An economic evaluation comparing the additional costs of an intervention with its additional health benefit, expressed in quality-adjusted life-years. CUAs inform whether incremental gains justify incremental costs.
Perspective	The analytic viewpoint that determines which costs and outcomes are included in the evaluation. This study used a US payer perspective, which excludes indirect costs such as productivity losses.
QALY	A measure combining the quantity and quality of life, where 1 QALY represents 1 year lived in perfect health. QALYs are used to quantify health outcomes in CUAs.
ICER^a^	A ratio representing the additional cost per additional QALY gained by 1 intervention compared with another.
Dominant choice	An intervention that is more effective (eg, yields more QALYs) and less costly than the comparator, and is therefore the preferred option.
WTP threshold	The maximum amount a health system or entity is willing to spend for 1 additional QALY. Interventions with ICERs below the threshold are generally considered cost-effective. A threshold of $50 000 per QALY is commonly used in the US.
INMB^b^	A monetary measure of cost-effectiveness calculated by converting incremental QALYs into monetary units using a specified WTP threshold and subtracting incremental costs. A positive INMB indicates that the intervention yields net value and is cost-effective at the chosen threshold.
Transition probability	The probability of moving from one health state to another during a given cycle of a Markov model.
Health utility	A numerical value (0 to 1) reflecting an individual’s preference for a specific health state, where 0 represents death and 1 represents perfect health.
Direct medical costs	Expenditures directly associated with the material or human resources required for disease management. This may include medication, hospitalization, outpatient visits, or other health care services.

Abbreviations: CUA, cost-utility analysis; ICER, incremental cost-effectiveness ratio; INMB, incremental net monetary benefit; QALY, quality-adjusted life-year; WTP, willingness-to-pay.

^a^ ICER = (early cost − delayed cost)/(early QALY − delayed QALY).

^b^ INMB = (ΔQALYs × WTP) − Δcosts.

### Data Sources and Input Parameters

A targeted literature review (TLR) of Medline (2000-2025) conducted by 2 independent reviewers (S.H. and J.C.) identified studies for 5 domains: clinical outcomes, EuroQol 5-Dimension questionnaire (EQ-5D) utilities, mortality, resource use and costs, and economic evaluations. Inclusion criteria, Patient/Population, Intervention, Comparison, and Outcome framework, and search strategy are in eTable 1 and eTable2 in Supplement 1.

### Target Population and Intervention

The modeled population comprised adult patients with clinically active SLE, defined by an SLE Disease Activity Index 2000 (SLEDAI-2K) score greater than 0, who had not previously received biologic therapy. Eligible patients met the 1982 revised American College of Rheumatology or 2012 Systemic Lupus International Collaborating Clinics classification criteria for SLE.^11,12^ The base case simulated 2 cohorts initiating intravenous belimumab (10 mg/kg at weeks 0, 2, and 4, and then every 4 weeks): an early initiation group (within 2 years of diagnosis) and a delayed initiation group (following failure of standard immunosuppressants).

### Model Structure and Health States

**Figure 1.**
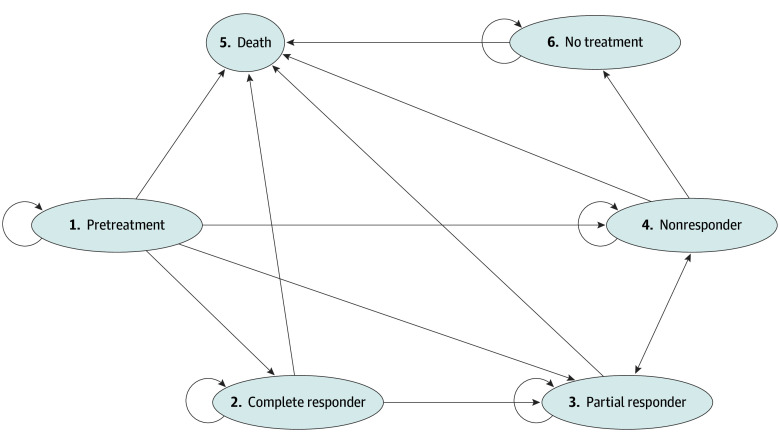
Markov Model State-Transition Diagram Diagram showing the 6 health states and allowable transitions in the Markov model evaluating early vs delayed belimumab initiation in systemic lupus erythematosus. Arrows indicate possible monthly transitions, with death as an absorbing state.

A state-transition Markov model was developed in TreeAge Pro Healthcare 2025 (TreeAge LLC) to simulate SLE disease course over a 15-year horizon, using monthly cycles. The model included 6 mutually exclusive health states: pretreatment, complete response, partial response, nonresponse, no treatment, and death (absorbing). All patients began in the pretreatment state and transitioned into 1 of the 3 response states after a 4-month induction period. Transitions followed a bidirectional stepwise structure, with movement from complete to partial responders and from partial to nonresponders. Nonresponders could discontinue belimumab, with pathways that included restarting, stopping, or switching therapy. Switchers were assumed to initiate anifrolumab, consistent with clinical practice.^13^ The state-transition diagram is provided in Figure 1.

Health states were defined using validated clinical instruments. Complete responders met the SLE Responder Index-4 (SRI-4) criteria (defined in eTable 3 in Supplement 1).^14^ Nonresponders failed to meet the SRI-4 criteria and either discontinued biologic therapy due to inefficacy or adverse events, or experienced a severe flare defined by the Safety of Estrogens in Lupus Erythematosus National Assessment–SLEDAI Flare Index (SELENA-SLEDAI; increase to >12).^15^ Patients with clinical improvement but not meeting SRI-4 and without evidence of treatment failure were classified as partial responders.

State-specific costs and utilities were applied identically across treatment groups to ensure that observed differences reflected transition patterns rather than state valuation, consistent with prior SLE cost-effectiveness analyses.^16^ Nonresponders accrued the highest costs and lowest utilities, reflecting greater morbidity and treatment escalation.^17^

### Transition Probabilities

Transition probabilities were derived from real-world cohort data demonstrating higher SRI-4 response rates with early disease.^8^ Discontinuation due to inefficacy or adverse events, severe flare rates, and SRI-4 over time were informed by the 13-year belimumab continuation study.^18^ Flare risk in the no treatment state was informed by a UK study reporting outcomes after belimumab withdrawal.^19^ Patients who flared in this state were assumed to initiate secondary therapy with anifrolumab. Anifrolumab transition probabilities were based on pooled phase III Treatment of Uncontrolled Lupus via the Interferon Pathway (TULIP)–1 and TULIP-2 data.^20,21^ Estimates were further calibrated to reflect biologic-experienced patients using outcomes from a post hoc TULIP analysis.^22^

### Mortality

All-cause mortality was modeled using US life-table probabilities adjusted by age- and sex-specific standardized mortality ratios from a large Italian SLE cohort, given the absence of comparable US standardized mortality ratio estimates.^23,24^ Model-generated mortality patterns were reviewed against published US epidemiologic data to confirm consistency with established SLE mortality trends.^25^

### Health Utilities

Health state utility values were drawn from a Swedish population-based study correlating EQ-5D scores with disease activity.^26^ Longitudinal EQ-5D data from an incident Swedish SLE cohort were used to anchor time-varying utility trends, with relative differences across health states applied to these trajectories.^27^

A validated glucocorticoid-related disutility was incorporated using an EQ-5D decrement of –0.005 per 1 mg per day increase in prednisone equivalent dose, applied to the difference in dose between cycles.^27^ Changes in prednisone use were informed by a prospective Chinese cohort of newly diagnosed, refractory, or relapsing SLE patients receiving belimumab.^9^ Prednisone tapering for anifrolumab was derived from the TULIP trials.^28^

### Costs and Resource Use

Costs were estimated from a US payer perspective and stratified by health state. Components included drug acquisition, hospitalizations, outpatient care, and emergency visits. Costs were converted to 2024 USD using the Consumer Price Index. Future costs and health effects were discounted at 3% annually.

Belimumab (400 mg per 5 mL) and anifrolumab (300 mg per 2 mL) were valued using January 2025 US wholesale acquisition cost (WAC) data.^29^ Secondary direct medical costs before and after belimumab initiation were derived from a US claims analysis of SLE patients initiating belimumab.^30^ Changes in secondary costs over time were informed by a Medicaid claims study describing longitudinal SLE cost trajectories, and differences across health states were taken from a large US study reporting expenditures by SLE disease severity.^31,32^ In the absence of analogous data for anifrolumab, secondary costs were assumed to be equivalent across biologics.

### Outcome Measures

Primary economic outcomes were the incremental cost-effectiveness ratio (ICER) and incremental net monetary benefit (INMB), using a willingness-to-pay (WTP) threshold of $50 000 per quality-adjusted life-year (QALY). The INMB quantified the WTP-adjusted net economic value, facilitating interpretation of cost-effectiveness and uncertainty across analyses.

### Statistical Analysis

#### Primary Analysis

Analyses were conducted in TreeAge Pro Healthcare 2025 and Excel version 16.103.3 (Microsoft). Model inputs were parameterized from the TLR using reported summary statistics to derive distributions for probabilistic sensitivity analyses. Beta distributions were applied to probabilities and utility values, log-normal distributions to costs and odds ratios (OR), and normal distributions to continuous clinical parameters.

#### Sensitivity Analyses

Probabilistic sensitivity analyses were performed using 10 000 Monte Carlo simulations in TreeAge, varying all parameters according to their prespecified distributions. 95% uncertainty intervals (UIs) for incremental costs, QALYs, and INMB were calculated from the 2.5 and 97.5 percentiles of the probabilistic sensitivity analyses distributions. Deterministic 1-way sensitivity analyses were conducted across model parameters, using published ranges or a ±30% variation when unavailable. Scenario analyses included a lifetime horizon and 2 biosimilar pricing scenarios. First, hypothetical biosimilar market entry was modeled as reductions in originator WACs based on US estimates of postbiosimilar price erosion.^33^ The second modeled biosimilar substitution using 15% and 60% WAC discounts, assuming equivalent efficacy and safety.^34^

## Results

### Targeted Literature Review

Demographic and clinical characteristics were aligned with the primary studies informing transition probabilities, yielding a cohort of 1000 patients (912 female [91.2%]) with a mean (SD) age of 41 (11) years at belimumab initiation. Early initiators had higher baseline disease activity (3 points higher SLEDAI-2K score) and glucocorticoid use (10 mg per day more of prednisone) relative to delayed initiators. The TLR identified 62 articles (eFigure 1 in Supplement 1). Six studies provided transition probability inputs,^8,18,19,20,21,23^ 4 studies informed utility estimates,^26,27,28,35^ and 3 studies provided cost data.^30,31,32^ No published economic evaluations comparing early vs delayed belimumab initiation were identified. Model parameters, distributions, sources, and key assumptions are summarized in eTable 3 in Supplement 1. Studies informing base case inputs were published between 2013 and 2025 and appraised using the Risk of Bias in Non-Randomized Studies of Interventions tool (eTable 4 in Supplement 1).

### Base Case Analysis

Over a 15-year horizon, early belimumab initiation accrued total discounted costs of $1 910 438.61 and 7.68 QALYs vs $2 036 775.73 and 7.38 QALYs for delayed initiation. Early initiation provided an additional 0.30 (95% UI, –0.42 to 1.39) QALYs at an incremental cost of –$126 337.12 (95% UI, –$910 010.39 to $168 383.94) per patient. This produced a favorable ICER of –$421 123.73 per QALY, below the WTP threshold of $50 000 per QALY.

### Probabilistic Sensitivity Analysis

**Figure 2.**
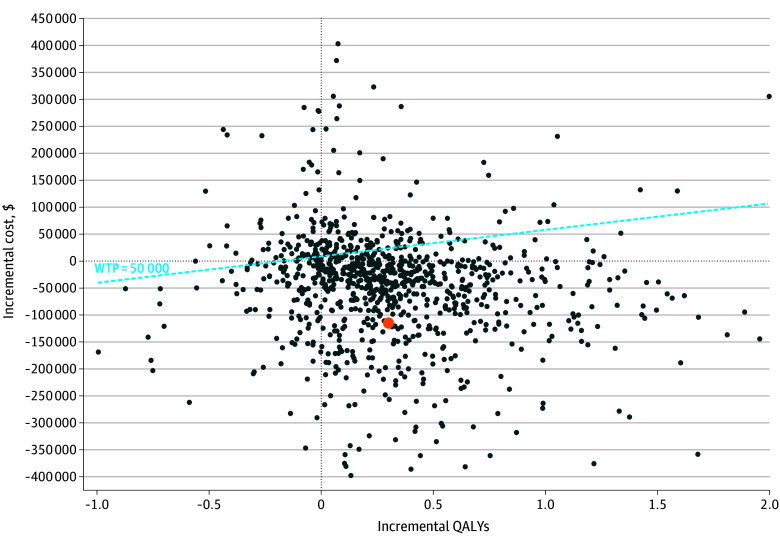
Incremental Cost-Effectiveness Scatterplot Each point represents a probabilistic sensitivity analysis iteration comparing early vs delayed belimumab initiation in systemic lupus erythematosus. The x-axis shows incremental quality-adjusted life years (QALYs) gained with early initiation, and the y-axis shows incremental costs ($). The dashed vertical and horizontal lines represent no incremental difference in effectiveness and cost, respectively. The diagonal dashed line represents the willingness-to-pay (WTP) threshold of $50 000 per QALY. Points falling below this line indicate simulations in which early initiation is cost-effective relative to delayed initiation. The orange point represents the base case estimate.

The incremental cost-effectiveness plane (Figure 2) was derived from 10 000 probabilistic simulations. The majority of simulations (6530 simulations [65.3%]) clustered in the southeast quadrant, indicating lower costs and higher QALYs for early initiation. The remaining simulations were distributed around the origin and across other quadrants, reflecting joint uncertainty in costs and health outcomes over the analytic horizon, with the central tendency favoring early initiation.

**Figure 3.**
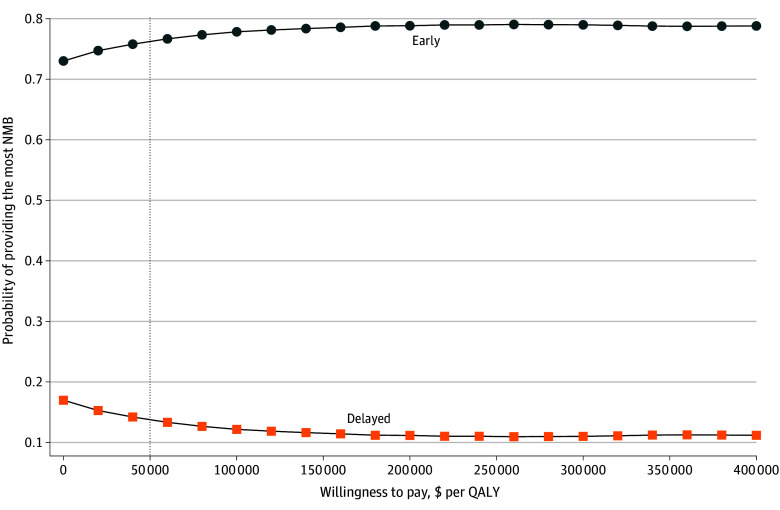
Cost-Effectiveness Acceptability Curve Cost-effectiveness acceptability curve showing the probability that early or delayed belimumab initiation is the most cost-effective strategy across varying willingness-to-pay thresholds, based on 10 000 probabilistic Monte Carlo simulations. NMB indicates net monetary benefit; QALY, quality-adjusted life-year.

The cost-effectiveness acceptability curve (Figure 3) demonstrates the probability that each strategy maximizes NMB across WTP thresholds. At a WTP of $50 000 per QALY, the mean INMB was $141 337.12 (95% UI, –$157 997.53 to $925 019.51), with early initiation favored in 8125 simulations (81.3%) vs 1875 simulations (18.8%) for delayed initiation. Early initiation remained more likely to maximize NMB across the evaluated WTP range.

### Deterministic Sensitivity Analyses

**Figure 4.**
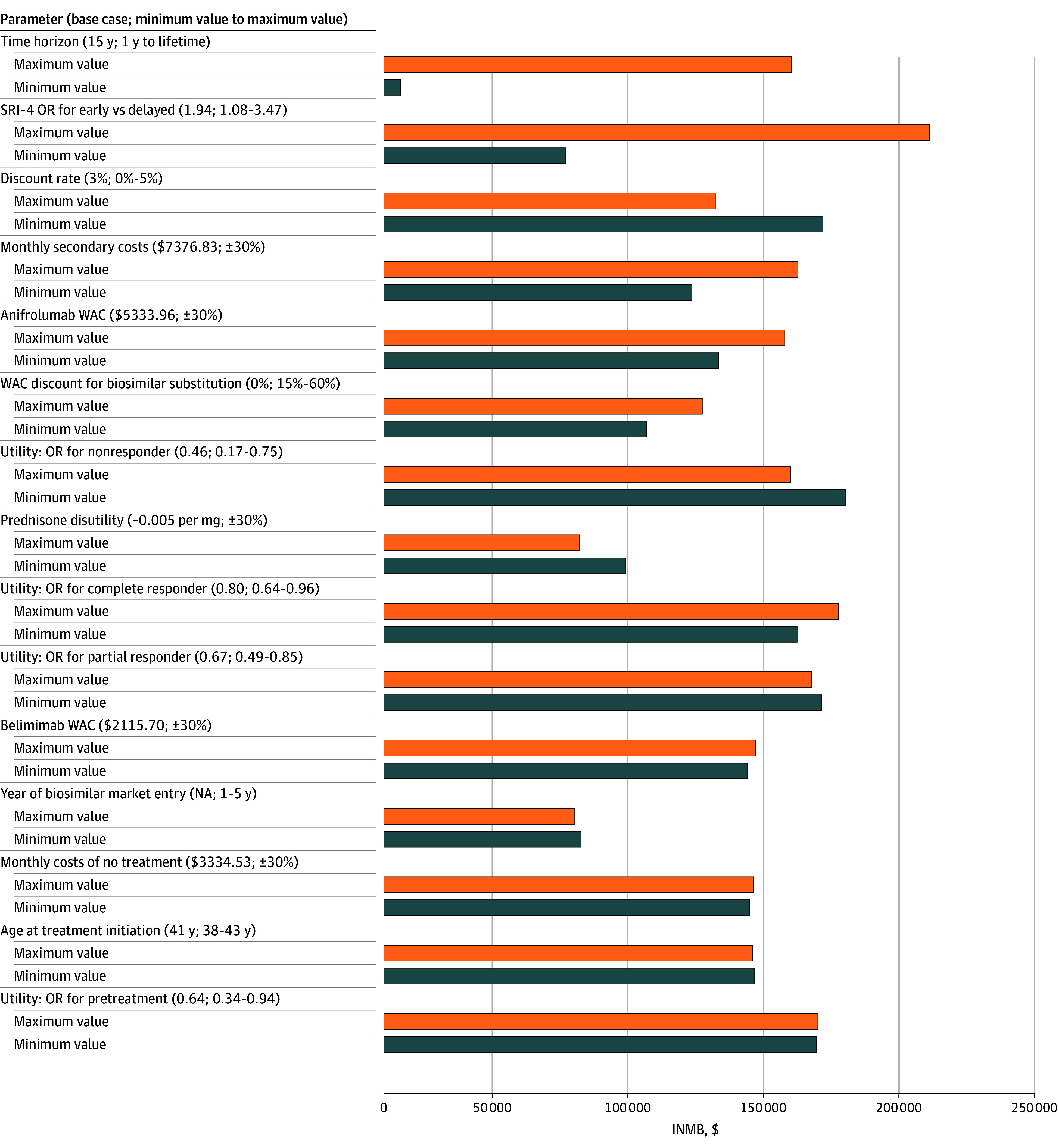
One-Way Sensitivity Analysis of Incremental Net Monetary Benefit Deterministic 1-way sensitivity analysis showing the incremental net monetary benefit (INMB) of early vs delayed belimumab initiation for varying key parameters. Parameters are listed on the vertical axis with their minimum and maximum values, and the horizontal axis reflects INMB in 2024 US dollars. Positive values indicate that early initiation is more cost-effective at a willingness-to-pay threshold of $50 000 per quality-adjusted life year (QALY). Bars represent INMB when each parameter is set to its minimum (blue) or maximum (orange) value. NA indicates not applicable; OR, odds ratio; SRI-4 indicates SLE Responder Index-4; WAC, wholesale acquisition cost.

One-way deterministic sensitivity analyses evaluating uncertainty in model parameters on the INMB at a WTP of $50 000 per QALY are summarized in the tornado diagram (Figure 4). The model was most sensitive to time horizon (INMB range, $6351.07 for a 1-year horizon to $156 497.12 for a lifetime horizon), SRI-4 response OR (INMB range, $69 741.68 for an OR of 1.08 to $209 591.09 for an OR of 3.47), and discount rate (INMB range, $127 597.15 at 0% to $168 764.76 at 5%). The highest INMB ($209 591.09) occurred under the maximum SRI-4 response OR, and the lowest ($6351.07) under a 1-year time horizon.

Outcome-specific, 1-way sensitivity analyses for incremental costs and incremental QALYs are presented in eFigure 2 and eFigure 3 in Supplement 1, respectively. Consistent with the INMB summary findings in Figure 4, variation in time horizon, SRI-4 response OR, and discount rate were the primary factors associated with uncertainty across both cost and health outcome measures.

INMBs across time horizons from 1 to 25 years are presented in eFigure 4 in Supplement 1. The INMB was $71 683.31 at 5 years and $141 337.14 at 15 years, with more gradual increases thereafter, reaching $156 497.12 at 25 years.

Scenario analyses examining alternative pricing assumptions showed that hypothetical biosimilar market entries at years 1 and 5 produced INMBs of $75 793.75 and $73 423.41, respectively. Biosimilar substitution modeled as 15% and 60% reductions to the WAC of the biologics resulted in INMBs of $100 997.04 and $122 353.56, respectively.

## Discussion

In this economic evaluation using cost-utility analysis to compare early vs delayed belimumab for clinically active SLE, early initiation generated greater health benefit and lower total medical costs, yielding positive INMBs across analyses. In the base case, early initiation produced a gain of 0.30 QALYs and cost savings of $126 337.12 per patient over 15 years, yielding a favorable ICER of –$421 123.73 per QALY and an INMB of $141 337.12 at a WTP threshold of $50 000 per QALY. Although the incremental QALY gain was modest, sustained utility improvements combined with substantial reductions in downstream costs generated a clear economic advantage.

Sensitivity analyses demonstrated that the results were robust across wide parameter ranges. The model was most sensitive to time horizon, SRI-4 response OR, and discount rate, yet the INMB remained positive throughout. Scenario analyses evaluating biosimilar entry and substitution also resulted in positive INMBs, indicating sustained economic favorability across a range of plausible future pricing environments. Probabilistic sensitivity analyses produced wide 95% UIs, reflecting uncertainty accumulating over a long horizon and correlation among time-varying parameters. Despite this breadth, early initiation remained the preferred strategy in more than 80% of simulations, demonstrating robust economic advantage across joint parameter uncertainty.

Differences in economic outcomes were due to the distribution of patients across health states. Early initiation led to faster and more sustained achievement of response states, which carried higher utility and lower secondary expenditures. Comparatively, delayed initiation left more patients in the nonresponder state, increasing cumulative costs and reducing QALY gains. These effects intensified over longer time horizons, with progressively larger INMBs as cumulative differences in cost and QALYs widened. Reduced time spent in costly, low-utility nonresponder states mitigated both short-term consequences of uncontrolled disease (eg, flares requiring hospitalization or therapy escalation) and longer-term risks (eg, irreversible organ damage from prolonged glucocorticoid exposure). A 15-year horizon was selected to reflect the chronicity of SLE while avoiding speculative lifetime extrapolations. In our model, the early cohort had higher baseline SLEDAI-2K scores and prednisone doses, which may increase SRI-4 response probabilities through regression to the mean, but also reflect a clinical context in which biologic responsiveness may be higher and irreversible damage has not yet accrued. Early belimumab initiation is therefore likely to be most cost-effective for newly diagnosed patients with higher disease activity, limited baseline organ damage, and greater glucocorticoid use at treatment initiation.^36^

Belimumab is less effective in patients with preexisting organ damage.^37^ Although organ damage was not modeled as a discrete state, its downstream effects were indirectly captured through response-specific prednisone trajectories and a validated prednisone-related EQ-5D decrement.^9,27^ Responders demonstrated greater glucocorticoid tapering, whereas nonresponders and delayed initiators remained on higher doses for longer. Because cumulative damage is tied to uncontrolled inflammation and prolonged glucocorticoid exposure, this structure captured the principal factors underlying long-term QALY loss without introducing an organ-damage module that would require unsupported transition rates or risk double-counting within the utility framework.^3,37^ This approach is conservative because prolonged glucocorticoid exposure would be expected to accrue more irreversible damage than the model fully reflects.

While this analysis focused on belimumab, our findings raise broader implications for SLE management. Emerging evidence from other immune-mediated diseases suggests that early biologic intervention may be disease-modifying, improving long-term outcomes and economic value.^38,39^ In SLE, earlier suppression of B-cell hyperactivity may also limit epitope spreading, where autoreactive immune responses expand to target additional autoantigens over time.^40^ This process promotes the formation of pathogenic immune complexes that drive organ complications, including glomerulonephritis, vasculitis, and central nervous system involvement.^40^ Interrupting this cascade early may preserve organ function, reduce irreversible damage, and alter disease trajectory. Given the high cumulative burden of flares and end-organ damage in SLE, future research should explore whether similar cost-effectiveness profiles apply to other targeted therapies when used early in the disease course. Comparative modeling across biologics, with stratification by baseline damage and disease activity, may help define optimal initiation timing for diverse patient subgroups.

### Strengths and Limitations

The key strength of this study was the rigor applied in model development, including a formal TLR, structured risk-of-bias assessments, and parameter inputs aligned with recent real-world SLE cohorts. Cross-validation was limited by the absence of prior economic evaluations examining timing of belimumab initiation, although this gap underscores the relevance of the present analysis.

This study also has limitations. First, several key inputs were derived from multinational rather than exclusively US cohorts. Belimumab effectiveness estimates came from international studies, and long-term utility trajectories and glucocorticoid-related disutility were taken from a Swedish cohort, while excess SLE-related mortality was modeled using standardized mortality ratios from an Italian population-based study. This implicitly assumes that treatment effects, utility trajectories, and relative mortality risks are broadly comparable across high-income settings. However, regional differences in health care policies, cost structures, and utility value sets may influence model generalizability. Patient-level factors including racial group, socioeconomic status, and comorbidities, may also influence both response to therapy and resource use, potentially impacting the preferred strategy.^41,42^

Second, inherent Markov model limitations, including memoryless assumption of disease states and fixed cycle lengths, may not fully capture the heterogeneous and fluctuating nature of SLE progression.^43^ Although cost and utility values were stratified by health state, they were applied uniformly across cohorts. This assumes that all patients in a given health state incur similar costs and comparable utility, which may not hold true in practice. For example, partial responders to early belimumab may require fewer adjunctive therapies and experience fewer subclinical flares than partial responders to delayed belimumab.

Third, the model assumed continuous treatment for responders across both cohorts, without accounting for potential differences in discontinuation due to sustained remission. Early responders are more likely to achieve durable disease control and may be candidates for treatment tapering or discontinuation.^35,44,45^ However, no robust data are currently available to quantify this effect. Additionally, data on concomitant hydroxychloroquine use were unavailable, despite evidence that hydroxychloroquine augments belimumab efficacy and reduces renal flares.^46^ Differential use of hydroxychloroquine between cohorts could influence clinical outcomes and downstream cost-effectiveness.

Collectively, these limitations suggest that the base case findings likely represent a conservative estimate of cost-effectiveness. They also highlight the need for further studies to capture more granular differences in utilities, response trajectories, and health care utilization between early and delayed belimumab users in contemporary US SLE populations.

## Conclusions

In this economic evaluation of early vs delayed initiation of belimumab in biologic-naive adults with clinically active SLE, early belimumab was both more effective and less costly than delayed initiation, rendering it the dominant strategy. These findings support reconsideration of current reimbursement criteria and advocate for earlier therapeutic intervention in appropriate patients. Given emerging evidence across immune-mediated diseases that early biologic treatment may be associated with more durable responses and less cumulative organ damage, cost-effectiveness modeling should be extended to other targeted therapies in SLE. A paradigm shift toward earlier use of disease-modifying agents may ultimately yield improved patient outcomes and greater long-term value for health systems.

## Notes

Supplement 1. eTable 1. Search Strategy eTable 2. PICO Framework eTable 3. Model Parameters, Distributions, and Assumptions eFigure 1. PRISMA Flow Diagram eTable 4. ROBINS-I Assessment eFigure 2. One-Way Sensitivity Analysis of Incremental Costs eFigure 3. One-Way Sensitivity Analysis of Incremental QALYs eFigure 4. Trajectory of Incremental Net Monetary Benefit Over Time eReferences

Supplement 2. Data Sharing Statement
