# Supplementary material for: Cost-Effectiveness of Early vs Delayed Belimumab Treatment for Systemic Lupus Erythematosus
Source: JAMA Netw Open. 2026 Feb 19;9(2):e2560167. doi: 10.1001/jamanetworkopen.2025.60167 (PMC12921522; doi:10.1001/jamanetworkopen.2025.60167)
Supplement: Supplement 1. — eTable 1. Search Strategy eTable 2. PICO Framework eTable 3. Model Parameters, Distributions, and Assumptions eFigure 1. PRISMA Flow Diagram eTable 4. ROBINS-I Assessment eFigure 2. One-Way Sensitivity Analysis of Incremental Costs eFigure 3. One-Way Sensitivity Analysis of Incremental QALYs eFigure 4. Trajectory of Incremental Net Monetary Benefit Over Time eReferences [file jamanetwopen-e2560167-s001.pdf]

Supplemental Online Content

Hundal S, Cappelli J, Sjöwall C, et al. Cost-effectiveness of early vs delayed belimumab treatment for systemic lupus erythematosus. *JAMA Netw. Open*. 2026;9(2):e2560167. doi:10.1001/jamanetworkopen.2025.60167

eTable 1. Search Strategy
eTable 2. PICO Framework
eTable 3. Model Parameters, Distributions, and Assumptions
eFigure 1. PRISMA Flow Diagram
eTable 4. ROBINS-I Assessment
eFigure 2. One-Way Sensitivity Analysis of Incremental Costs
eFigure 3. One-Way Sensitivity Analysis of Incremental QALYs
eFigure 4. Trajectory of Incremental Net Monetary Benefit Over Time
eReferences

This supplemental material has been provided by the authors to give readers additional information about their work.

© 2026 Hundal S et al. *JAMA Network Open*.

eTable 1. Search Strategy

**Area of Interest**	**Search Strategy**	**Hits**
Intervention & Comparator Specific Data		
	("systemic lupus erythematosus"[MeSH Terms] OR "SLE"[All Fields]) AND (("prednisone"[All Fields] OR "oral glucocorticoid"[All Fields]) OR ("biologics"[All Fields] OR "belimumab"[All Fields] OR "anifrolumab"[All Fields]))	2939
Clinical Outcomes	AND ("flare"[All Fields] OR "recurrence"[All Fields] OR "complications"[All Fields] OR "adverse events"[All Fields] OR "side effects"[All Fields] OR "remission"[All Fields])	1611
Utilities	OR ("EQ-5D"[All Fields] OR "utility"[All Fields] OR "utility weight"[All Fields])	1631
Economic Models	OR ("cost utility"[All Fields] OR "cost benefit"[All Fields] OR "cost effectiveness"[All Fields] OR "cost"[All Fields])	1639
Resource Use	OR ("resource utilization"[All Fields] OR "healthcare utilization"[All Fields] OR "hospitalization"[All Fields] OR "emergency department"[All Fields] OR "economic burden"[All Fields] OR "outpatient visit"[All Fields] OR "mortality"[All Fields] OR "death"[All Fields]))	1664
Limitations	AND English[lang] AND ("2000/01/01"[PDAT] : "2025/11/30"[PDAT])	965

© 2026 Hundal S et al. *JAMA Network Open*.

eTable 2. PICO Framework

	Inclusion	Exclusion
Population	- Patients aged ≥ 18 years<br>- Diagnosis of SLE, according to ACR or SLICC criteria<br>- Clinically active disease (SLEDAI > 0)	- Previous exposure to other B-cell targeted therapies<br>- Pregnancy or breastfeeding<br>- Life-threatening kidney or CNS involvement
Intervention (Early)	- IV Belimumab 10 mg/kg on week 0, 2, 4, then every 4 weeks)<br>- Initiated within 2 years of disease duration	- Belimumab used for non-SLE indications or in combination with other biologics
Comparator (Delayed)	- IV Belimumab 10 mg/kg on week 0, 2, 4, then every 4 weeks)<br>- Initiated for refractory disease, after failure of standard of care	- Belimumab used for non-SLE indications or in combination with other biologics
Intervention & Comparator Outcomes	- Response rates<br>- Flare rates<br>- Adverse event rates<br>- Discontinuation rates<br>- Improvement rates<br>- Healthcare resource utilization	- If outcomes of interest are not reported<br>- Immunologic or molecular outcomes<br>- Outcomes focused on treatments other than the intervention or comparator
SLE Outcomes	- Utility weight, EQ-5D index score<br>- Mortality rate (all-cause)<br>- Direct medical costs for the healthcare system	- If outcomes of interest are not reported<br>- Outcomes for conditions other than SLE
Study Design	- Systematic reviews, meta-analyses<br>- RCT, cohort studies, case-control studies<br>- Economic papers or utility values involving SLE patients<br>- English language only<br>- Studies published in the last 25 years (2000 – 2025)	- Narrative reviews, commentaries, opinions, editorials, letters without primary data<br>- Published in languages other than English<br>- Studies focused on conditions other than SLE<br>- Studies focused on SLE comorbidities<br>- Qualitative studies

Abbreviations: EQ-5D, EuroQOL-5 dimensions; SLE, systemic lupus erythematosus.

© 2026 Hundal S et al. *JAMA Network Open*.

eTable 3. Model Parameters, Distributions, and Assumptions

Parameter	Base Case (cycle)	Range Over Time	Distribution	Source	Assumptions
Utilities					
Baseline EQ-5D index	0.64 (cycle 1)	0.57-0.71	Beta	[1]	Mean baseline EQ-5D for clinically active SLE was used as the initial utility for all patients before biologic treatment. These values were taken from a Swedish longitudinal cohort, which is the only source providing robust long-term EQ-5D trajectories in SLE. Although the Swedish value set was applied in a U.S. context, this was considered appropriate because EQ-5D changes associated with SLE disease activity are biologically mediated rather than health-system dependent, and no comparable U.S. longitudinal data exist. The observed trajectory was used to model utility changes over time and to define the no treatment health state. Utilities varied over the first eight years to reflect observed patterns and then held constant due to limited long-term data.
Utility multiplier: complete responder (CR)	1.25	Fixed	Log normal	[2]	EQ-5D in the group with the lowest Systemic Lupus Activity Questionnaire (SLAQ) scores (score 0-9; mean 0.80) was divided by the overall cohort mean EQ-5D (0.64) to derive a multiplicative CR utility ratio of 1.25. Non-U.S., Swedish data. Although this study included participants with self-reported SLE rather than physician-verified diagnoses, utilities were used solely to inform relative differences across health states rather than to establish disease prevalence or severity distributions, reducing susceptibility to diagnostic misclassification. Assumes relative utility gain in low-activity vs average SLE is similar in U.S. SLE. Multipliers are applied to the time-varying baseline EQ-5D, so the model preserves relative differences between health states rather than absolute Swedish utilities.
Utility multiplier: partial responder (PR)	1.05	Fixed	Log normal	[2]	EQ-5D in the intermediate SLAQ group (score 10-15; mean 0.67) was divided by the overall cohort mean EQ-5D (0.64) to derive a multiplicative PR utility ratio of 1.05.
Utility multiplier: non- responder (NR)	0.72	Fixed	Log normal	[2]	EQ-5D in the highest SLAQ group (score 16-37; mean 0.46) was divided by the overall cohort mean EQ-5D (0.64) to derive a multiplicative NR utility ratio of 0.72.
Prednisone disutility per 1 mg/day (EQ-5D units)	-0.005	Fixed	Beta	[1]	The EQ-5D decrement per mg/day of prednisone was taken from regression analyses in the Swedish longitudinal cohort, in which daily glucocorticoid dose was an independent predictor of EQ-5D. This marginal disutility was assumed to be applicable to U.S.

© 2026 Hundal S et al. *JAMA Network Open*.

Parameter	Base Case (cycle)	Range Over Time	Distribution	Source	Assumptions
					patients because the adverse effects of glucocorticoids are biologically driven rather than system-specific. The decrement was applied additively to health-state utilities each monthly cycle according to the modeled prednisone dose.
Glucocorticoid Parameters					
Prednisone change in responders (early)	-30 mg/d (cycle 3)	-30 to +0.05	Normal	[3]	Mean change in daily prednisone dose among newly diagnosed SLE patients starting belimumab in a Chinese real-world cohort was used to parameterize the initial glucocorticoid-sparing effect. This dataset was selected because no equivalent U.S. longitudinal data describe prednisone tapering trajectories stratified by treatment timing and response. Non-U.S. data was deemed acceptable as the biologic effect of belimumab on glucocorticoid requirements was assumed to generalize across settings. Monthly changes were interpolated from visit-level data up to 18 months, assumed to converge with delayed belimumab between 18 months and 4 years, then held constant.
Prednisone change in responders (delayed)	-1.25 (cycle 3)	-3.66 to +0.05	Normal	[3]	The delayed belimumab responder trajectory was derived from the refractory subgroup of the same Chinese cohort, reflecting attenuated glucocorticoid-sparing in patients treated later in the disease course. These data were applied to U.S. patients on the assumption that timing of biologic initiation, rather than specific health-system factors, primarily drives differences in glucocorticoid reduction. Monthly changes were modeled to 18 months, aligned with early-treatment trajectories to year 4, then constant.
Prednisone change in non-responders (early)	0 (cycle 3)	0 to +4.5	Normal	[3]	Estimated from the difference in prednisone trajectories between the newly diagnosed and relapsing subgroups in the Chinese cohort, with the relapsing subgroup used as a proxy for non-response or flare. These non-U.S. data were applied to the U.S. setting under the assumption that inadequate disease control results in comparable glucocorticoid requirements across populations, as this relationship is biologically rather than system dependent. Monthly prednisone changes were interpolated from visit-level data through 18 months, assumed to align with early responder trajectories between 18 months and 4 years, and then held constant due to limited long-term evidence.

© 2026 Hundal S et al. *JAMA Network Open*.

Parameter	Base Case (cycle)	Range Over Time	Distribution	Source	Assumptions
Prednisone change in non-responders (delayed)	+11.25 (cycle 3)	3.13 to 11.25	Normal	[3]	Estimated from the difference in prednisone trajectories between the "refractory" and "relapsing" subgroups in the Chinese cohort, with the relapsing subgroup serving as a proxy for non-response or flare. These non-U.S. data were applied to the U.S. setting under the assumption that inadequate disease control results in comparable glucocorticoid requirements across populations. Monthly prednisone changes were interpolated from visit-level data through 18 months, assumed to align with delayed responder trajectories between 18 months and 4 years, then held constant.
Costs (2024 USD)					
Belimumab WAC (per 400mg / 5mL)	$2115.7	Fixed	Log normal	[4]	Belimumab unit cost was derived from January 2025 U.S. wholesale acquisition cost (WAC) for the 400 mg IV vial and applied according to the modeled dosing schedule (induction at weeks 0, 2, and 4, then by monthly). This price was assumed constant in real terms over the time horizon, consistent with standard base-case HTA practice. No rebates or price erosion were included. Hypothetical biosimilar discounts and alternative pricing scenarios were examined only in scenario analyses.
Anifrolumab WAC (per 300mg / 2mL)	$5333.96	Fixed	Log normal	[4]	Anifrolumab unit cost was derived from January 2025 U.S. WAC for a 300 mg IV dose, administered every 4 weeks. This price was assumed constant in real terms over the time horizon, consistent with standard HTA practice. No rebates or price erosion were included. Alternative pricing examined in scenario analyses.
Monthly baseline secondary direct medical costs	$7376.83	Fixed	Log normal	[5]	Annual all-cause medical costs for U.S. patients initiating IV belimumab were obtained from commercial and Medicare claims and converted to a monthly cost excluding belimumab drug costs. These secondary costs were assumed to capture ongoing SLE-related care and comorbidities and were applied to both cohorts, as well as anifrolumab, with the assumption that background resource use is similar across biologics. Costs were inflated to 2024 USD and varied over time using a separate time-trend factor.
Cost of no treatment health state	$3334.53	Fixed	Log normal	[5]	Pre-index annual costs from SLE patients prior to belimumab initiation were used to estimate the resource use associated with standard of care treatment. These values were converted to a monthly cost and inflated to 2024 USD. The resulting estimate was applied whenever patients were not receiving biologic therapy, including the period before treatment initiation (pre-treatment) and the no treatment state after discontinuation.

© 2026 Hundal S et al. *JAMA Network Open*.

Parameter	Base Case (cycle)	Range Over Time	Distribution	Source	Assumptions
Cost inflation / time trend factor	1 (cycle 1)	1-1.28	Log normal	[6]	Relative changes in annual medical costs over five years for SLE patients without lupus nephritis were extracted from the U.S. Medicaid claims analysis. These annual cost increases were converted into a multiplicative time-trend factor to adjust secondary costs in the model, capturing the rise in healthcare utilization and expenditures as disease accumulates over time. The trend was applied through year 5 and then held constant due to absence of reliable longer-term data.
Cost ratio: CR	0.66 (cycle 1)	0.6-0.77	Log normal	[7]	Ratios of annual costs in U.S. patients with mild SLE relative to the overall SLE population were used to represent lower secondary costs in CR health state. Mild disease in the claims-based severity algorithm was assumed to approximate patients in durable complete response. The ratio was applied multiplicatively to secondary costs each cycle, allowed to vary through year 4, then held constant due to limited long-term severity-stratified data.
Cost ratio: PR	1.11 (cycle 1)	0.98-1.16	Log normal	[7]	The moderate-to-overall SLE cost ratio from the same U.S. claims study was mapped to the PR health state, reflecting modestly higher resource use when disease control is incomplete. The ratio was applied multiplicatively to the secondary costs each cycle, allowed to vary through year 4, and then held constant.
Cost ratio: NR	1.86 (cycle 1)	1.48-1.86	Log normal	[7]	The severe-to-overall SLE cost ratio, reflecting substantially higher annual expenditures, was applied to the NR health state. Severe disease in the claims-based algorithm was treated as a proxy for non-response, capturing patients with persistently active or uncontrolled SLE who incur higher resource use. The ratio was applied multiplicatively to the secondary costs each cycle, allowed to vary through year 4, and then held constant.
Belimumab Clinical Inputs					
Initial Health-State Assignments					
SRI-4 response OR (early versus delayed)	1.94 (cycle 4)	1.94-3.79	Log-normal	[8]	SLE Responder Index-4 (SRI-4) response odds ratio (OR) for early versus delayed belimumab initiation was taken from an Italian multicenter real-world cohort with 36 months of follow-up. Although non-U.S., these data were used because they directly evaluate timing of belimumab initiation in routine care, which is not available in U.S. datasets. The relative effect of early versus delayed initiation was assumed transferable to the U.S., where underlying SLE physiology and belimumab dosing are similar.

© 2026 Hundal S et al. *JAMA Network Open*.

Parameter	Base Case (cycle)	Range Over Time	Distribution	Source	Assumptions
					SRI-4 response requires a ≥4-point reduction in Safety of Estrogens in Lupus Erythematosus National Assessment (SELENA)-SLEDAI, no significant deterioration in the Physician's Global Assessment (PGA; <0.3-point increase in PGA from baseline), no new British Isles Lupus Assessment Group (BILAG) A and not more than one new BILAG B domain score.[9]
Transition to CR at 4 months (early)	45.07%	Fixed	Beta	10	CR entry at 4 months was calibrated using SRI-4 rates from the multinational long-term belimumab extension study, adjusted by the early versus delayed initiation odds ratio reported by Gatto et al. This calibration ensured that the combined early and delayed cohorts reproduced the overall SRI-4 response observed in the extension study while maintaining the relative advantage of early initiation.<br><br>SRI-4 response at 16 weeks was assumed to correspond to entry into the CR health state, as CR in the model is defined on the basis of SRI-4 achievement. CR probabilities were held constant during the induction period and then incorporated into the monthly transition matrix for subsequent cycles.
Transition to CR at 4 months (delayed)	30.4%	Fixed	Beta	10	
Monthly Transition Probabilities					
PR → CR or NR → PR (early)	3.55%	Fixed	Beta	8,10	Monthly transitions among PR, CR, and NR were estimated by fitting regression models to SRI-4 response trajectories from the multinational belimumab extension program, which included North American, European, and Asia-Pacific sites. These fitted trajectories were converted into monthly transition probabilities capturing improvement (e.g., PR→ CR) and loss of response (e.g., CR→ PR). Transitions were assumed applicable to U.S. practice because belimumab dosing, monitoring intervals, and response assessment criteria are standardized across regions, and SLE disease activity patterns do not vary meaningfully by system.<br><br>Transition probabilities were held constant within prespecified post-induction intervals and calibrated such that the simulated longitudinal response curves reproduced observed SRI-4 patterns while preserving the relative benefit of early initiation as quantified by the SRI-4 OR.
PR → CR or NR → PR (delayed)	2.67%	Fixed	Beta	8,10	
CR → PR (early)	1.09%	Fixed	Beta	8,10	
CR → PR (delayed)	1.59%	Fixed	Beta	8,10	

© 2026 Hundal S et al. *JAMA Network Open*.

Parameter	Base Case (cycle)	Range Over Time	Distribution	Source	Assumptions
Event Rates					
Lack of efficacy leading to discontinuation	0.03%	0-0.18%	Beta	[10]	Discontinuation rates due to lack of efficacy were reported for >11 years of follow-up in the long-term belimumab extension study. These year-specific rates were converted to monthly probabilities and applied equally to early and delayed belimumab cohorts. A single pooled monthly rate was assumed across health states because discontinuation for lack of efficacy was consistently low and did not meaningfully vary over time or by disease severity. Patients discontinuing for lack of efficacy were assumed to either switch to anifrolumab or discontinue biologic therapy entirely in equal proportions (50/50), reflecting the absence of empirical data on post-belimumab treatment pathways.
Adverse event requiring discontinuation	0.06%	0-0.28%	Beta	[10]	Discontinuation rates due to adverse events were also reported for each year across more than 11 years of follow-up in the long-term belimumab extension study. These year-specific values were converted to monthly probabilities and applied uniformly to both early and delayed belimumab cohorts. A pooled monthly adverse event-related discontinuation rate was used because adverse event profiles remained stable over time and did not materially differ by treatment duration or disease severity. Patients discontinuing due to adverse events were assumed to either transition to anifrolumab or discontinue biologic therapy in equal proportions (50/50), given the lack of empirical data on post-adverse event treatment patterns.
Severe flare rate	0.25%	0-0.83%	Poisson	[10]	Severe flare rates were taken from year-specific severe flare incidences reported across >11 years in the multinational long-term belimumab extension study, as defined by the SELENA-SLEDAI Flare Index. These annual rates were converted to monthly probabilities using a Poisson process, assuming flares occur independently and at a constant rate within each year. The resulting time-varying monthly probabilities were applied to both cohorts, as long-term evidence does not indicate differential flare risk once patients are established on treatment. The year 11+ flare rate was carried forward thereafter due to limited longer-term data. Patients experiencing a severe flare were modeled to either switch to anifrolumab or discontinue therapy in equal proportions (50/50). Severe flares were assumed to accrue additional costs (captured as NR) but not to independently modify long-term mortality risk.

© 2026 Hundal S et al. *JAMA Network Open*.

Parameter	Base Case (cycle)	Range Over Time	Distribution	Source	Assumptions
No treatment state flare rate	33.3%	Fixed	Beta	[11]	Flare risk in the no-treatment health state was derived from the BEL116027 open-label withdrawal study, which reported flare incidence over 24 weeks following cessation of belimumab. These data were used to parameterize monthly flare probabilities for patients not receiving biologic therapy. Although multinational, the cohort was considered applicable to U.S. practice because flares after biologic withdrawal reflect underlying SLE disease activity rather than health-system factors. Patients experiencing a severe flare in the no treatment state were assumed to start anifrolumab, representing clinical re-escalation of therapy after loss of control.
Mortality rate	0.10%	0.08-0.34%	Beta	[12,13]	Age- and sex-specific U.S. mortality rates from national life tables were adjusted using age- and sex-specific standardized mortality ratios (SMRs) from a large Italian population-based SLE cohort. SMRs were applied separately for women and men and mapped to the modeled cohort assuming a starting age of 41 years and a sex distribution of 91% female and 9% male, consistent with contemporary SLE trial populations.<br><br>Although the SMRs were estimated outside the U.S., they were considered applicable because the relative excess mortality from SLE reflects underlying disease biology and damage accrual rather than health-system factors, and available data suggest similar SMR patterns across high-income settings. SMR-adjusted mortality rates were applied equally across all cohorts, as there is no evidence of differential mortality risk between early versus delayed belimumab or between belimumab and anifrolumab.
**Anifrolumab Inputs**					
Prednisone change in responders	-3.59 (cycle 13)	-3.59 to +0.05	Normal	[14]	Prednisone requirements for anifrolumab were estimated from the TULIP glucocorticoid taper analysis, which compared cumulative prednisone in responders and non-responders. These data were applied to represent prednisone trajectories for CR and NR in the model and were considered transferable to the U.S. setting because glucocorticoid use is driven by biologic disease control rather than health-system factors. Monthly prednisone changes were derived through 52 weeks, aligned with belimumab-based trajectories beyond one year for comparability, and held constant after four years due to limited longer-term evidence.
Prednisone change in non-responders	-4.78 (cycle 13)	-4.78 to +0.05	Normal	[14]	

© 2026 Hundal S et al. *JAMA Network Open*.

Parameter	Base Case (cycle)	Range Over Time	Distribution	Source	Assumptions
Transition to CR at 1 month	9.25%	Fixed	Beta	[15,16]	Derived from pooled TULIP-1 and TULIP-2 SRI-4 response rates, with an adjustment factor applied to reflect biologic-experienced patients in post-hoc analyses. These multinational trial data, including North American participants, were assumed applicable to U.S. The same CR probability was used irrespective of prior belimumab timing once patients initiated anifrolumab due to lack of data stratifying response based on treatment initiation timing.
PR → CR or NR → PR	11.71%	Fixed	Beta	[15,16]	Monthly transitions among PR, CR, and NR were estimated from pooled SRI-4 response trajectories in the TULIP trials. Fitted responder curves were converted into monthly probabilities reflecting improvement and loss of response. Because patients entering anifrolumab in the model have failed belimumab, these base probabilities were adjusted using the relative reduction in SRI-4 response observed in biologic-experienced versus biologic-naive patients in the pooled TULIP subgroup analysis.
CR → PR	10.18%	Fixed	Beta	[15,16]	
Lack of efficacy	0.09%	Fixed	Beta	[17]	Discontinuation due to lack of efficacy was based on the annual discontinuation rate reported in the pooled TULIP trials. This was converted to a monthly probability and applied uniformly to all patients receiving anifrolumab. Because subgroup-specific discontinuation estimates were not available for biologic-experienced versus biologic-naive patients, a single pooled rate was used rather than stratified values. Lack of efficacy rates were assumed to be independent of current health state or duration of treatment, reflecting limited evidence to support time- or state-dependent variation. Patients discontinuing for lack of efficacy were assumed to transition to the no treatment health state.
Adverse event requiring discontinuation	0.46%	Fixed	Beta	[15]	Adverse event-related discontinuation for anifrolumab was derived from the biologic-experienced subgroup in the pooled post hoc TULIP analysis. This annual probability was converted to a monthly discontinuation rate and applied uniformly to all patients receiving anifrolumab after switching from belimumab, reflecting the model's assumption that all anifrolumab initiators are biologic-experienced. Adverse event-related discontinuation was assumed independent of current health state or treatment duration due to limited evidence supporting time or state varying risks. Patients discontinuing due to adverse events were assumed to enter the no-treatment health state.

© 2026 Hundal S et al. *JAMA Network Open*.

Parameter	Base Case (cycle)	Range Over Time	Distribution	Source	Assumptions
Flare rate	5.30%	Fixed	Beta	[15]	Flare risk for anifrolumab was derived from the annualized overall flare rate reported for biologic-experienced patients in the pooled post hoc TULIP analysis. The annual flare rate was converted to a monthly flare probability and applied uniformly to all anifrolumab recipients. Severe flare or SFI specific rates were not reported in the TULIP trials. Therefore, a single overall flare probability was used, with any flare assumed to represent loss of disease control consistent with transition to the NR state. Flare risk was modeled as independent of treatment timing and duration due to the absence of evidence supporting differential or time-dependent flare patterns in anifrolumab.

Abbreviations: CR, complete responder; EQ-5D, EuroQOL-5 dimensions; PR, partial responder; NR, non-responder; USD$, United States Dollar; US, United States; PsA, probabilistic sensitivity analysis; SRI-4, SLE Responder Index-4; SLAQ, Systemic Lupus Activity Questionnaire; OR; odds ratio.

© 2026 Hundal S et al. *JAMA Network Open*.

eFigure 1. PRISMA Flow Diagram.

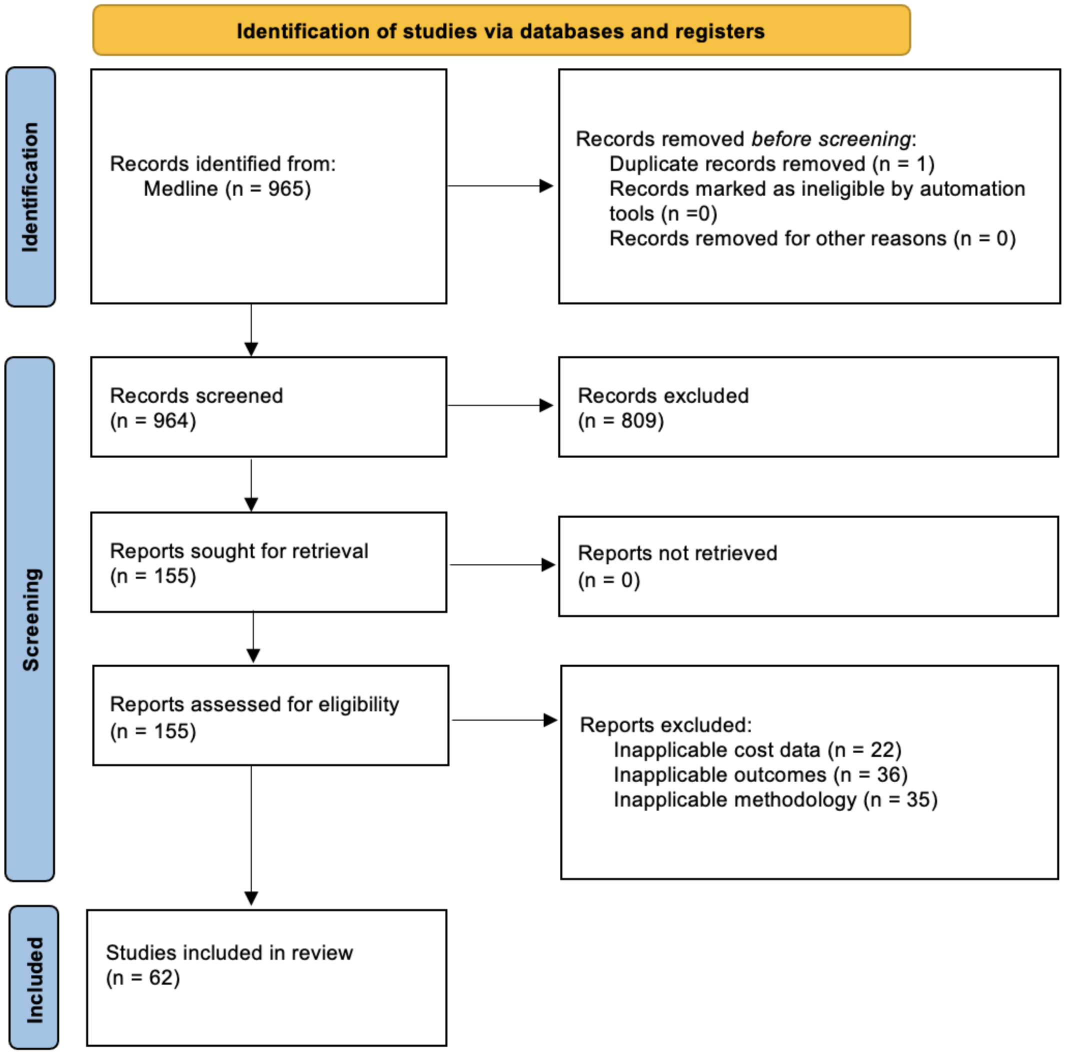

© 2026 Hundal S et al. *JAMA Network Open*.

eTable 4. ROBINS-I Assessment

Study (Author, Year)	D1	D2	D3	D4	D5	D6	D7	Overall
Parodis et al., 2025[1]	Low	Low	Low	Low	Low	Low	Low	Low
Bexelius et al., 2013[2]	Mod*	Mod*	Low	Low	Mod*	Low	Mod*	Mod*
Zhao et al., 2025[3]	Mod*	Mod*	Low	Low	Mod*	Mod*	Low	Mod*
Lokhandwala et al., 2021[5]	Mod*	Mod*	Low	Mod*	Low	Mod*	Mod*	Mod*
Li et al., 2009[6]	Mod*	Low	Low	Low	Low	Low	Low	Mod*
Murimi-Worstell et al., 2021[7]	Mod*	Mod*	Mod*	Low	Mod*	Low	Low	Mod*
Gatto et al., 2020[8]	Mod	Low	Low	Low	Low	Low	Low	Mod*
Wallace et al., 2019[10]	Mod*	Mod*	Low	Mod*	Mod*	Mod*	Low	Mod*
Bae et al., 2022[11]	Mod*	Mod*	Low	Mod*	Low	Mod*	Low	Mod*
Zen et al., 2023[12]	Mod*	Low	Low	Low	Low	Low	Low	Mod*
Bruce et al., 2023[14]	Mod*	Low	Low	Low	Low	Low	Low	Mod*
Morand et al., 2020[17]	Low	Low	Low	Low	Low	Low	Low	Low

*Moderate

Legend. D1: bias due to confounding, D2: bias due to selection of participants, D3: bias in classification of interventions, D4: bias due to deviations from intended interventions, D5: bias due to missing data, D6: bias in measurement of outcomes, D7: bias in selection of the reported result.

© 2026 Hundal S et al. *JAMA Network Open*.

eFigure 2. One-Way Sensitivity Analysis of Incremental Costs

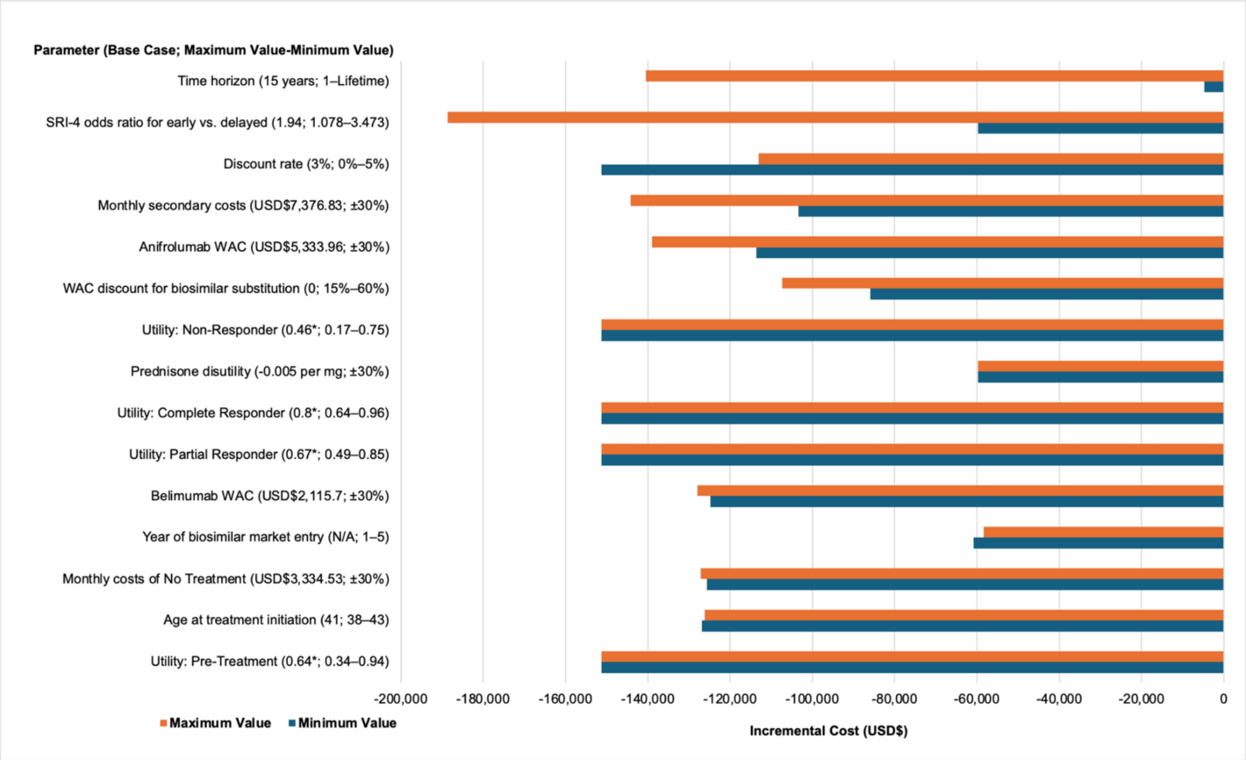

Legend. One-way sensitivity analysis showing the impact of varying key model parameters on the incremental direct medical costs of early versus delayed belimumab initiation. Parameters are displayed on the vertical axis with their associated minimum and maximum values, and the horizontal axis reflects incremental cost in 2024 USD. Negative values indicate that early initiation is less costly than delayed initiation. Bars represent incremental costs when parameters are set to their minimum (blue) and maximum (orange) values.

Abbreviations: QALY, quality-adjusted life-year; SRI-4, SLE Responder Index-4; USD$, United States Dollar; WAC, wholesale acquisition cost.

© 2026 Hundal S et al. *JAMA Network Open*.

eFigure 3. One-Way Sensitivity Analysis of Incremental QALYs

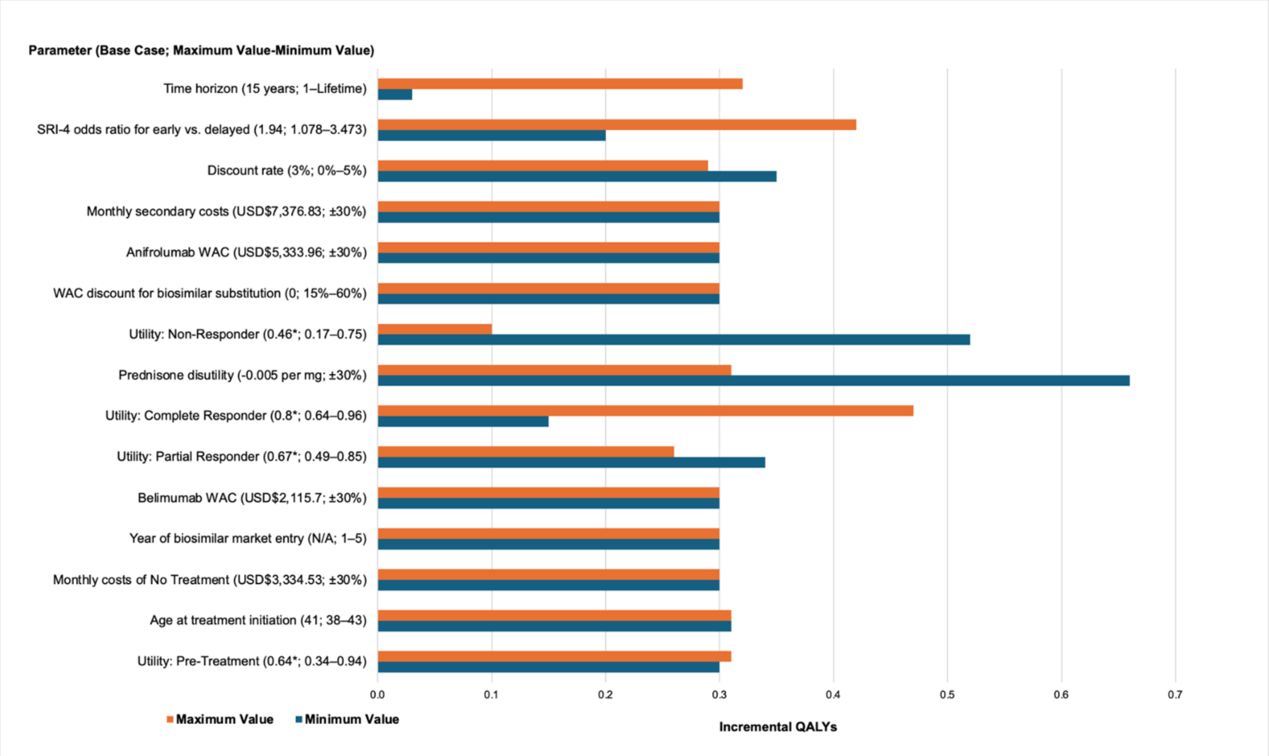

Legend. One-way sensitivity analysis showing the impact of varying key model parameters on the incremental quality-adjusted life-years (QALYs) of early versus delayed belimumab initiation. Parameters and their minimum and maximum values are shown on the vertical axis. The horizontal axis displays incremental QALYs. Bars represent incremental QALYs when parameters are set to their minimum (blue) and maximum (orange) values, demonstrating the parameters contributing most to uncertainty in effectiveness.

Abbreviations: QALY, quality-adjusted life-year; SRI-4, SLE Responder Index-4; USD$, United States Dollar; WAC, wholesale acquisition cost.

© 2026 Hundal S et al. *JAMA Network Open*.

eFigure 4. Trajectory of Incremental Net Monetary Benefit Over Time

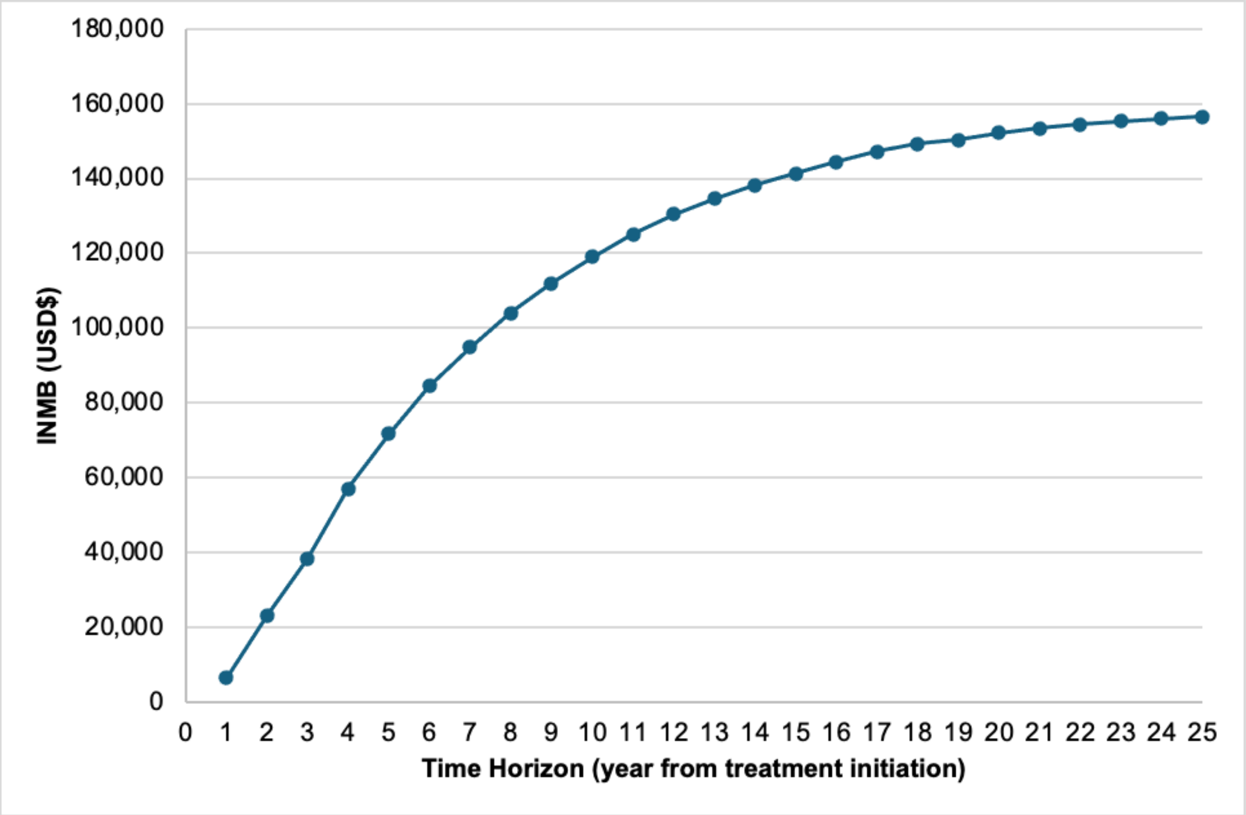

Legend. Incremental net monetary benefit (INMB) of early versus delayed belimumab initiation evaluated at cumulative time horizons from 1 to 25 years. The curve depicts the INMB at each horizon, reflecting how the net monetary benefit changes as costs and health outcomes accrue over longer durations.

Abbreviations: INMB, incremental net monetary benefit; USD$, United States Dollar.

© 2026 Hundal S et al. *JAMA Network Open*.

eReferences

1. Parodis I, Haugli-Stephens T, Dominicus A, Eek D, Sjöwall C. Lupus Low Disease Activity State and organ damage in relation to quality of life in systemic lupus erythematosus: a cohort study with up to 11 years of follow-up. *Rheumatology (Oxford)*. 2025;64(2):639-647.

2. Bexelius C, Wachtmeister K, Skare P, Jönsson L, van Vollenhoven R. Drivers of cost and health-related quality of life in patients with systemic lupus erythematosus (SLE): a Swedish nationwide study based on patient reports. *Lupus*. 2013;22(8):793-801.

3. Zhao Y, Qi F, Bai J, et al. Real-world efficacy of belimumab in systemic lupus erythematosus: a prospective cohort from a single centre in China. *Rheumatology (Oxford)*. 2025;64(1):108-116.

4. HCPCS Drug Pricing Search Tool. Accessed December 2, 2025. https://pricing.buyandbill.com/

5. Lokhandwala T, Coutinho AD, Bell CF. Retrospective analysis of disease severity, health care resource utilization, and costs among patients initiating belimumab for the treatment of systemic lupus erythematosus. *Clin Ther*. 2021;43(8):1320-1335.

6. Li T, Carls GS, Panopalis P, Wang S, Gibson TB, Goetzel RZ. Long-term medical costs and resource utilization in systemic lupus erythematosus and lupus nephritis: a five-year analysis of a large medicaid population. *Arthritis Rheum*. 2009;61(6):755-763.

7. Murimi-Worstell IB, Lin DH, Kan H, et al. Healthcare utilization and costs of systemic lupus erythematosus by disease severity in the United States. *J Rheumatol*. 2021;48(3):385-393.

8. Gatto M, Saccon F, Zen M, et al. Early disease and low baseline damage as predictors of response to belimumab in patients with systemic lupus erythematosus in a real-life setting. *Arthritis Rheumatol*. 2020;72(8):1314-1324.

9. Furie RA, Petri MA, Wallace DJ, et al. Novel evidence-based systemic lupus erythematosus responder index. *Arthritis Rheum*. 2009;61(9):1143-1151.

10. Wallace DJ, Ginzler EM, Merrill JT, et al. Safety and efficacy of belimumab plus standard therapy for up to thirteen years in patients with systemic lupus erythematosus. *Arthritis Rheumatol*. 2019;71(7):1125-1134.

11. Bae SC, Bass DL, Chu M, et al. The effect of 24-week belimumab treatment withdrawal followed by treatment restart in patients with SLE: an open-label, non-randomised 52-week study. *Arthritis Res Ther*. 2022;24(1):46.

© 2026 Hundal S et al. *JAMA Network Open*.

12. Zen M, Salmaso L, Barbiellini Amidei C, et al. Mortality and causes of death in systemic lupus erythematosus over the last decade: Data from a large population-based study. *Eur J Intern Med*. 2023;112:45-51.

13. Actuarial Life Table. Accessed December 2, 2025. https://www.ssa.gov/oact/STATS/table4c6.html

14. Bruce IN, van Vollenhoven RF, Morand EF, et al. Sustained glucocorticoid tapering in the phase 3 trials of anifrolumab: a post hoc analysis of the TULIP-1 and TULIP-2 trials. *Rheumatology (Oxford)*. 2023;62(4):1526-1534.

15. Efficacy of Anifrolumab in Patients with SLE Previously Treated with Biologics: Post Hoc Analysis of Data from 2 Phase 3 Trials. ACR Meeting Abstracts. September 1, 2021. Accessed November 30, 2025. https://acrabstracts.org/abstract/efficacy-of-anifrolumab-in-patients-with-sle-previously-treated-with-biologics-post-hoc-analysis-of-data-from-2-phase-3-trials/

16. Bruce IN, van Vollenhoven RF, Psachoulia K, Lindholm C, Maho E, Tummala R. Time to onset of clinical response to anifrolumab in patients with SLE: pooled data from the phase III TULIP-1 and TULIP-2 trials. *Lupus Sci Med*. 2023;10(1):e000761.

17. Morand EF, Furie R, Tanaka Y, et al. Trial of anifrolumab in active systemic lupus erythematosus. *N Engl J Med*. 2020;382(3):211-221.

© 2026 Hundal S et al. *JAMA Network Open*.
