# Supplementary material for: Cost-Effectiveness of Early vs Delayed Belimumab Treatment for Systemic Lupus Erythematosus
Source: JAMA Netw Open. 2026 Feb 19;9(2):e2560167. doi: 10.1001/jamanetworkopen.2025.60167 (PMC12921522; doi:10.1001/jamanetworkopen.2025.60167)
Supplement: Supplement 2. — Data Sharing Statement [file jamanetwopen-e2560167-s002.pdf]

Data Sharing Statement

Hundal. Cost-Effectiveness of Early vs Delayed Belimumab Treatment for Systemic Lupus Erythematosus. *JAMA Netw Open*. Published February 19, 2026.
doi:10.1001/jamanetworkopen.2025.60167

Data
Data available: Yes
Data types: Data (not involving human participants)
How to access data: Provided via email from corresponding author
When available: With publication

Supporting Documents
Document types: Statistical/analytic code
How to access documents: Provided via email from corresponding author
When available: With publication

Additional Information
Who can access the data: Researchers whose proposed use of the data has been approved
Types of analyses: All inputs (parameters) and outputs from Markov models will be made available
Mechanisms of data availability: With investigator support, written request to first author via email
